# Editorial: (Osteo)Sarcopenia & sarcopenic obesity

**DOI:** 10.3389/fendo.2023.1270350

**Published:** 2023-08-24

**Authors:** Stefano Masiero, Maria Chiara Maccarone, Ifigenia Kostoglou-Athanassiou, Yannis Dionyssiotis

**Affiliations:** ^1^ Rehabilitation Unit, Department of Neuroscience, University of Padova, Padua, Italy; ^2^ Physical Medicine and Rehabilitation School, University of Padova, Padua, Italy; ^3^ Endocrinology Department, “Asclepeion” General Hospital, Voula, Greece; ^4^ Spinal Cord Injury Rehabilitation Clinic, General University Hospital Patras, Rio Patras, Greece

**Keywords:** aging, rehabilitation, frailty, osteopenia, osteoporosis

Worldwide, life expectancy and the number of elderly individuals are increasing due to social, health, and technological advancements (1–3). Therefore, public health systems and scientific communities should seriously consider the “pandemia” of conditions associated with advanced age (4). In recent years, significant progress has been made in understanding the mechanisms underlying sarcopenia and its implications, particularly in relation to changes in bone metabolism and obesity. It has been recognized that osteosarcopenia, a syndrome characterized by the concurrent presence of osteoporosis, sarcopenia, and altered body composition, shares common outcomes and therapeutic targets, and is expected to become more prevalent due to factors such as immunosenescence, sedentarism, obesity, and fat infiltration, contributing to a higher risk of frailty, institutionalization, falls, and fractures among the elderly (5). Furthermore, preliminary findings suggest that obesity and sarcopenia have synergistic effects on various health consequences (6).

Given the potential economic and social burden of these conditions, it is crucial to develop new strategies for proper clinical management (7). The main objective of the Research Topic “(Osteo)Sarcopenia & Sarcopenic Obesity” is to describe the clinical, biochemical, diagnostic, and functional aspects of osteosarcopenic and sarcopenic-obese patients in order to provide innovative tools for the management of these conditions.

So far, the diagnosis of sarcopenia and related conditions requires assessing muscle mass, muscle strength, and physical performance (2). One of the key aims in the near future is to establish screening and early diagnosis tools (8). Two original articles in our Research Topic have explored this aspect. Yin et al. conducted a cross-sectional analysis using baseline data from the West China Health and Aging Trend (WCHAT) study, investigating the relationship between various biomarkers and sarcopenia. The authors identified fasting insulin combined with the aspartate aminotransferase to alanine aminotransferase (AST/ALT) ratio as appropriate predictors of sarcopenia, suggesting the use of these biomarkers for early detection. Additionally, He et al. found that higher AST/ALT and lower insulin*prealbumin (INS*PA) product were associated with a higher prevalence of sarcopenia among elderly Chinese participants in the WCHAT study. The authors recommended regular monitoring of AST/ALT and INS*PA as a suitable approach for sarcopenia screening and management, given the affordability and simplicity of these blood biomarkers. Future studies should further explore the relationship between biochemical and metabolic markers and sarcopenia to develop more versatile and user-friendly screening tools.

Another topic of growing interest is the association between sarcopenia and other conditions, including inactivity, changes in endocrine function, inflammation, and chronic disorders. Veronese et al. conducted a longitudinal cohort study investigating the association of sarcopenia with radiographic and symptomatic knee osteoarthritis (OA). Over a four-year follow-up period, the presence of sarcopenia at baseline was strongly linked to a higher risk of developing symptomatic knee OA, while there was no significant difference in radiographic OA prevalence. Dionyssiotis et al. conducted a cross-sectional study involving hospitalized patients with neurodisabilities and healthy controls, and they observed significantly lower body mass index (BMI) and skeletal muscle index (SMI) scores in neurological patients. Similarly, elderly patients with musculoskeletal disorders exhibited a high prevalence of sarcopenia, and a correlation between sarcopenia and increased BMI has been found in this population (8).

In an observational study, Qiao et al. examined the association between the A Body Shape Index (ABSI) and/or sarcopenia and total, cardiovascular, and cancer mortality. The ABSI value, which assesses the health implications of body height, mass, and waist circumference, was found to be significantly associated with all-cause and cardiovascular mortality. Additionally, the coexistence of higher ABSI values and sarcopenia appeared to contribute to a higher risk of death compared to high ABSI values or sarcopenia alone.

Finally, given the substantial societal burden of age-related osteoporosis and sarcopenia and the evidence indicating their mutual influence, a study by Liu et al. within the Research Topic aimed to assess the causality between osteoporosis and sarcopenia using instrumental variables and Mendelian randomization. The results revealed a significant causal effect between osteoporosis and sarcopenia, with severe osteoporosis increasing the risk of appendicular lean mass loss and subsequent reduced lumbar spine bone mineral density (Liu et al.). These findings emphasize the importance of addressing both osteoporosis and sarcopenia in the prevention, diagnosis, and treatment of osteosarcopenia, providing valuable insights for patient management.

The results of the original studies in the Research Topic highlight the association between sarcopenia and various comorbidities, including neurological and musculoskeletal concerns as well as obesity, which can lead to adverse outcomes and increased mortality risk. Serological biomarkers show promise in the early detection of sarcopenia and should represent a valuable resource for clinical practice, allowing early detection of osteosarcopenia and sarcopenic obesity, and mitigating the progression of associated conditions.

By recognizing and diagnosing osteosarcopenia and osteopenic obesity, healthcare professionals can tailor interventions that address the specific needs of these individuals. This includes implementing exercise programs to improve muscle strength, bone density, and balance, as well as adopting nutritional strategies to support optimal musculoskeletal health (9, 10). For osteosarcopenia, treatment should include osteoporotic drugs, resistance and balance exercises, and nutritional recommendations (9).

A comprehensive review by Pahlavani reveals the potential of exercise therapy, particularly exercises with blood flow restriction, in reversing the decreased muscle mass and strength, increased adipose mass, and pro-inflammatory subset associated with sarcopenic obesity. Exercises with blood flow restriction may be recommended for individuals unable to engage in high-intensity strength training due to chronic illnesses and sarcopenic obesity (Pahlavani). Nevertheless, understanding osteosarcopenia and sarcopenic obesity is crucial for providing appropriate management strategies.

In conclusion, the journey towards a complete understanding of these conditions and their management remains challenging; the studies presented in this Research Topic have provided valuable insights into the complexity of sarcopenia-related conditions and the necessity for early diagnosis and personalized rehabilitation. The findings from the manuscripts included in the Research Topic underscore the importance of developing multidisciplinary approaches in the future. We extend our gratitude to all the participants and contributors to the research projects and the papers included in this Research Topic, with the hope that the information presented will advance clinical practice and inspire further innovative research in the future.

## Author contributions

SM: Writing – original draft, Writing – review & editing. MM: Conceptualization, Writing – original draft, Writing – review & editing. IK-A: Supervision, Writing – review & editing. YD: Conceptualization, Writing – review & editing.
